# CACNA1C (rs1006737) may be a susceptibility gene for schizophrenia: An updated meta‐analysis

**DOI:** 10.1002/brb3.1292

**Published:** 2019-04-29

**Authors:** Dongjian Zhu, Jingwen Yin, Chunmei Liang, Xudong Luo, Dong Lv, Zhun Dai, Susu Xiong, Jiawu Fu, You Li, Juda Lin, Zhixiong Lin, Yajun Wang, Guoda Ma

**Affiliations:** ^1^ Department of Psychiatry Affiliated Hospital of Guangdong Medical University Zhanjiang China; ^2^ Department of Neurology Affiliated Hospital of Guangdong Medical University Zhanjiang China; ^3^ Guangdong Key Laboratory of Age‐Related Cardiac and Cerebral Diseases Guangdong Medical University Zhanjiang China; ^4^ Clinical Research Center Affiliated Hospital of Guangdong Medical University Zhanjiang China

**Keywords:** CACNA1C, meta‐analysis, rs1006737, schizophrenia

## Abstract

**Introduction:**

Schizophrenia is a serious mental illness with a genetic predisposition. Genome‐wide association studies (GWAS) have identified the α‐1C subunit of the L‐type voltage‐gated calcium channel (*CACNA1C*) gene as a significant risk gene for schizophrenia. However, there are inconsistent conclusions in case–control studies.

**Methods:**

We performed a comprehensive meta‐analysis of all available samples from existing studies under four different genetic models (recessive model, dominant model, additive model and allele model) to further confirm whether *CACNA1C* rs1006737 is an authentic risk single nucleotide polymorphism (SNP) for schizophrenia.

**Results:**

A statistically significant difference under the four models (all *p* < 0.05) was observed by pooling nine Asian and European studies, including a total of 12,744 cases and 16,460 controls. For European‐decent samples, a significant difference was identified between patients and controls for the four models (all *p* < 0.05). We observed a significant difference between patients and controls for the recessive model and allele model (GG vs. GA + AA: *p* < 0.00001; G vs. A: *p* < 0.00001) using a fixed effect model, but the dominant model (GG + GA vs. AA: OR: *p* = 0.15) and additive model (GG vs. AA: *p* = 0.11) showed no significant difference between patients and controls in the Asian samples.

**Conclusion:**

Our findings provide important evidence for the establishment of *CACNA1C* as a susceptibility gene for schizophrenia across world populations, but its roles in the pathogenesis of schizophrenia need to be further investigated.

## 1 INTRODUCTION

Schizophrenia is a severe psychiatric disease that has a serious adverse impact on society, families, and patients, affecting approximately 1% of the worldwide population (Sukanta, David, Joy, & John, 2005). According to research, its heritability is as high as 80% (Sullivan, Kendler, & Neale, 2003). Recently, GWAS have identified the α‐1C subunit of the L‐type voltage‐gated calcium channel (*CACNA1C*) gene as a significant risk gene for schizophrenia (Gurung & Prata, 2015). Although CACNA1C was strongly associated with schizophrenia in previous studies, it is still unclear how it affects the onset of schizophrenia.

The *CACNA1C* gene, located on chromosome 12p13.3, encodes an α‐1 subunit of the L‐type voltage‐dependent gated calcium channel. This channel transiently increases the membrane permeability for calcium‐mediated cell membrane depolarization, playing an essential role in dendritic development, neuronal survival, synaptic plasticity, memory formation, learning, and behavior (Bhat et al., 2012). According to the neurodevelopmental hypothesis of schizophrenia (Fatemi & Folsom, 2009), any factor that can affect the development of the nervous system may be the cause of schizophrenia; thus, the *CACNA1C* gene may be involved in schizophrenia by regulating the development of the nervous system. In addition, *CACNA1C* rs1006737 has also shown significant associations with other mental illnesses, such as bipolar disorder and major depressive disorder (Ferreira et al., 2008; Green et al., 2010; Liu et al., 2011).

Based on the potential possibility of shared risk variants in schizophrenia, studies from Europe reported a significant association of the A‐allele of SNP rs1006737 with schizophrenia in a Danish cohort (Nyegaard et al., 2010), a British cohort (Green et al., 2010), and a Spanish cohort (Ivorra et al., 2014). These results were successfully replicated in some Asian studies (Guan et al., 2014; Guanchen, Zhang, Fuquan, Zhiqiang, & Wei, 2017; Kuanjun et al., 2014; Porcelli et al., 2015; Zheng et al., 2014). However, several studies from Pakistan, Japan and Shanghai, China, have failed to replicate the above results (Fatima et al., 2017; Hori et al., 2012; Zhang et al., 2012). Given the inconsistent association results, whether *CACNA1C* rs1006737 is associated with schizophrenia remains to be elucidated.

Meta‐analysis is a method for collecting, merging, and statistically analyzing different research results. Recently, Jiang et al. (2015), Zheng et al. (2014), and Nie, Wang, Zhao, Zhang, and Ma (2015) have conducted meta‐analysis combining Asian and European studies on the association between schizophrenia and rs1006737. However, the studies involved only one genetic model (allelic model). We therefore conducted a meta‐analysis integrating nine studies under four different genetic models to evaluate the association of rs1006737 in the *CACNA1C* gene with schizophrenia.

## 2 MATERIALS AND METHODS

### 2.1 Literature search

The PubMed, Web of Science, Cochrane Central Register of Controlled Trials, Science Direct, Wiley Online Library, Chinese National Knowledge Infrastructure, and WanFang Data databases were searched for potentially eligible studies using the combination of the keywords “CACNA1C,” “rs1006737,” and “schizophrenia,” with no limitations placed on language. All articles were evaluated on the basis of the title and abstract, and studies that were clearly irrelevant were excluded. Then, the full texts of potentially eligible studies were reviewed in full to determine the inclusion in the meta‐analysis.

### 2.2 Inclusion and exclusion criteria

Eligible studies in the meta‐analysis had to fulfill the following criteria: (a) evaluate the *CACNA1C* rs1006737 polymorphism in relation to schizophrenia; (b) consist of a human case–control study; (c) include patients meeting the diagnostic criteria for schizophrenia according to the Diagnostic and Statistical Manual of Mental Disorders, 4th ed. (DSM‐IV) or the International Classification of Diseases–10 (ICD‐10), with control participants having no history of mental disorders, other neurological disorders, and alcohol or drug abuse; (d) provide sufficient data for calculating the genotypic odds ratio (OR) with a 95% confidence interval (95% CI); (e) no overlap of samples with the other identified references; and (f) published before November 2018.

Studies with the following criteria were excluded from the current analysis: (a) not a case–control study; (b) duplicates of previous publications; (c) abstracts, comments, reviews, posters, and editorials; and (d) reports lacking detailed genotype data.

### 2.3 Data extraction

Data for this meta‐analysis were extracted using a standardized data extraction form independently by the authors. The following data were extracted from the eligible study: first author's name, year of publication, country of origin, ethnicity, sample techniques, number of cases and controls, Hardy–Weinberg equilibrium (HWE) score, and allele and genotype frequencies, among other information. If the authors did not provide additional information, the studies were excluded.

### 2.4 Statistical analysis

HWE was assessed for each study using the chi‐squared test. *p* > 0.05 was considered to be consistent with HWE. Meta‐analysis was performed using RevMan 5.3 software (RRID:SCR_00358, Cochrane). Pooled ORs (odds ratio) and their 95% CIs (95% confidence intervals) were calculated to assess the association between *CACNA1C* rs1006737 and susceptibility to schizophrenia for the recessive model (GG vs. AG + AA), dominant model (GG + AG vs. AA), additive model (GG vs. AA), and allele model (G vs. A). Pooled ORs with Z‐test *p* < 0.05 were considered statistically significant. Statistical heterogeneity among studies was assessed by Cochran's *Q*‐test and the *I* ^2^ metric. Cochran's *Q*‐test approximately follows a distribution with k‐1 degrees of freedom (k stands for the number of studies in the analysis). The *I* ^2^ metric was used and ranges from 0% to 100%. Low, moderate, large, and extreme heterogeneity corresponded to 0%–25%, 25%–50%, 50%–75% and 75%–100%, respectively. *p* < 0.05 and *I* ^2^ > 50% were deemed to indicate significant heterogeneity. A fixed effect model (Mantel–Haenszel method, M–H) was used in the absence of heterogeneity; otherwise, a random effect model (using the DerSimonian and Laird's method) was applied. Sensitivity analysis was performed to evaluate the influence of each study on the overall pooled result by sequentially excluding each individual study. A funnel plot was generated to evaluate the potential publication bias using Stata 15.1 software (RRID:SCR_007244, Stata Corp). Furthermore, power analysis was performed by Power and Sample Size Calculation software (RRID:SCR_004943, Dupont and Plummer).

## 3 RESULTS

### 3.1 Study inclusion and characteristics

**Figure 1:**
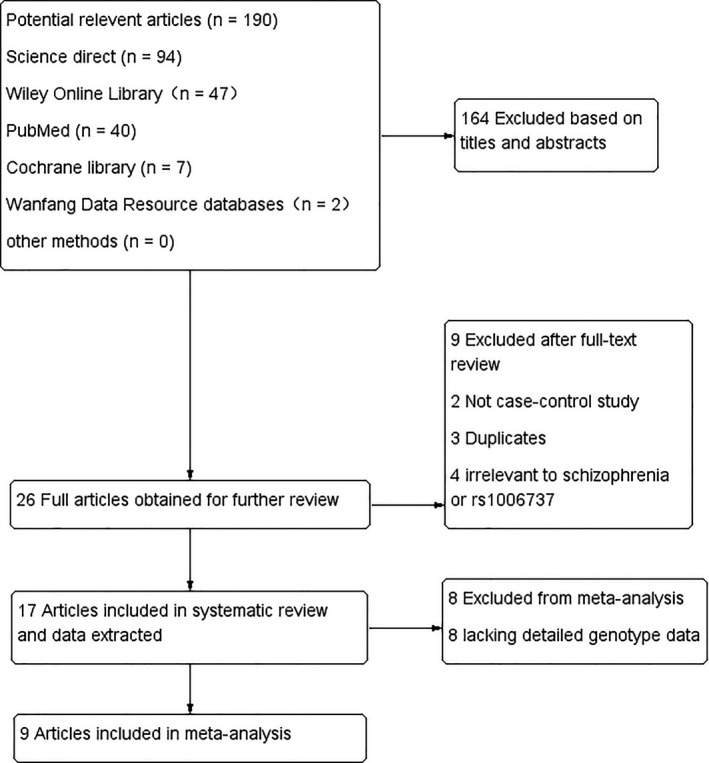
Flow diagram of the literature search and selection

A flow chart of the literature search and selection process is shown in Figure 1. A total of 190 potentially relevant articles were identified in the initial search. After screening the title and summary, 164 records were excluded. Thus, 26 published articles were retained. We then assessed the full texts and nine were excluded, among them two were not case–control studies, three were duplicates, and four were irrelevant to schizophrenia or rs1006737. Hence, 17 articles were included in the systematic review, but when data were extracted, eight studies lacked detailed genotype data and were excluded. Thus, nine studies (Fatima et al., 2017; Green et al., 2010; Guan et al., 2014; Guanchen et al., 2017; He et al., 2014; Hori et al., 2012; Nyegaard et al., 2010; Zhang et al., 2012; Zheng et al., 2014) were considered eligible for the present meta‐analysis.

The characteristics of each study are shown in Table 1. A total of 12,744 schizophrenia cases and 16,460 healthy controls were included in the present study. All the genotype distributions in each group were consistent with HWE.

**Table 1:** Characteristics of the studies included in the meta‐analysis

First author,	Published Year	Country	Ethnicity	Sample techniques	*N* (cases/controls)	HWE (P)	Cases				Controls			
							GG	GA	AA	MAF	GG	GA	AA	MAF
Ambrin Fatima	2017	Pakistan/Denmark	Pakistani	ABI 3130XL genetic analyzer	494/298	>0.05	393	84	17	0.119	235	54	9	0.121
EK Green	2010	UK	European	Affymetrix assay	479/2936	>0.05	205	208	66	0.355	1,367	1,233	336	0.324
Fanfan Zheng	2014	China	Chinese	TaqMan SNP genotyping assay	5,893/6319	>0.05	5,239	635	19	0.057	5,706	597	16	0.050
Fanglin Guan	2014	China	Chinese	The Sequenom MassARRAY	1,430/1570	>0.05	1,061	343	26	0.138	1,223	327	20	0.117
Gai Guanche	2017	China	Chinese	Unknown	1,372/1087	>0.05	1,229	140	3	0.053	1,003	80	4	0.041
Hiroaki Hori	2012	Japan	Japanese	TaqMan 59‐exonuclease allelic discrimination assay	552/1132	>0.05	480	70	2	0.067	1,002	127	3	0.059
Kuanjun He 2013)	2013	China	Chinese	TaqMan SNP Genotyping Assays	1,230/1228	>0.05	996	220	14	0.101	1,053	166	9	0.075
M Nyegaard	2010	Denmark	European	the Sequenom MassARRAY	976/1489	>0.05	402	444	130	0.361	656	675	158	0.333
Qiumei Zhang	2012	China	Chinese	Taqman allele‐specific assays	318/401	>0.05	280	37	1	0.061	357	42	2	0.057

We conducted a power analysis for detecting significant allelic associations; our total sample size and Asian sample size revealed a 100% power using OR values for the risk allele of 1.20，but the power for the European sample size was 83.1%.

### 3.2 Results of the overall meta‐analysis

**Figure 2:**
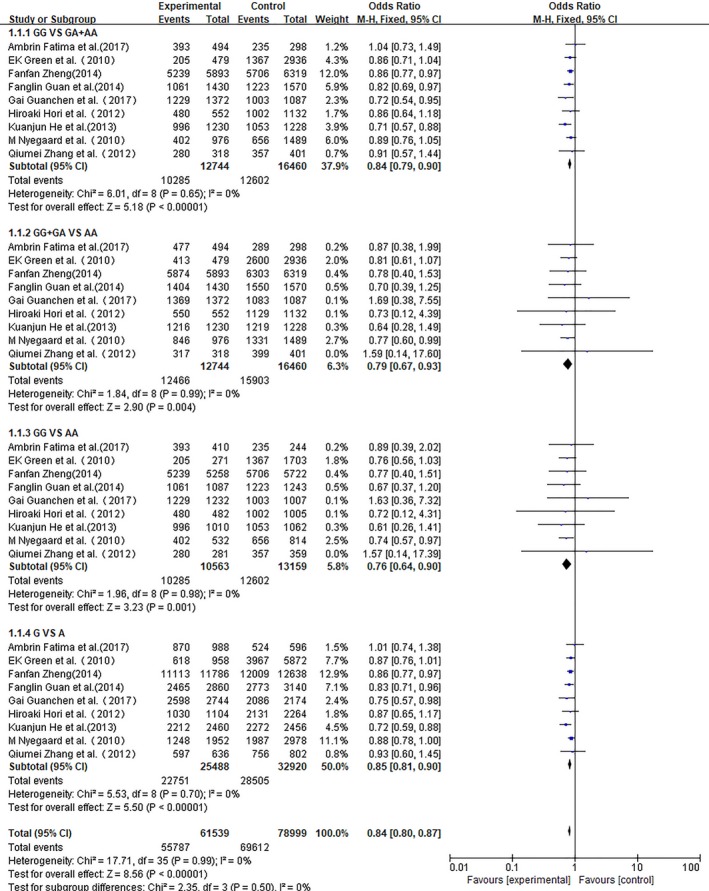
Meta‐analysis for the association of rs1006737 with schizophrenia in the European population and Asian population

**Figure 3:**
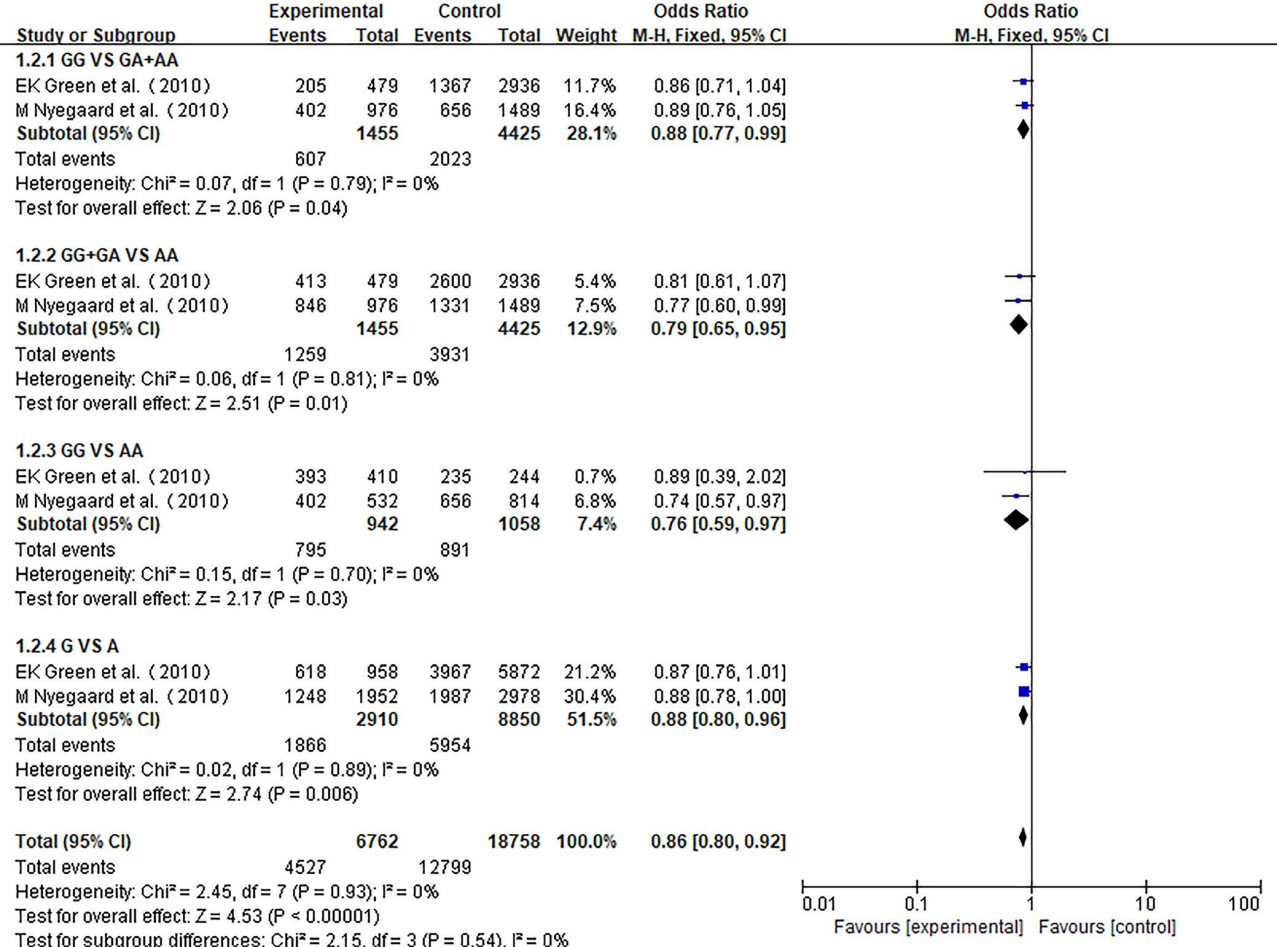
Meta‐analysis for the association of rs1006737 with schizophrenia in the European population

**Figure 4:**
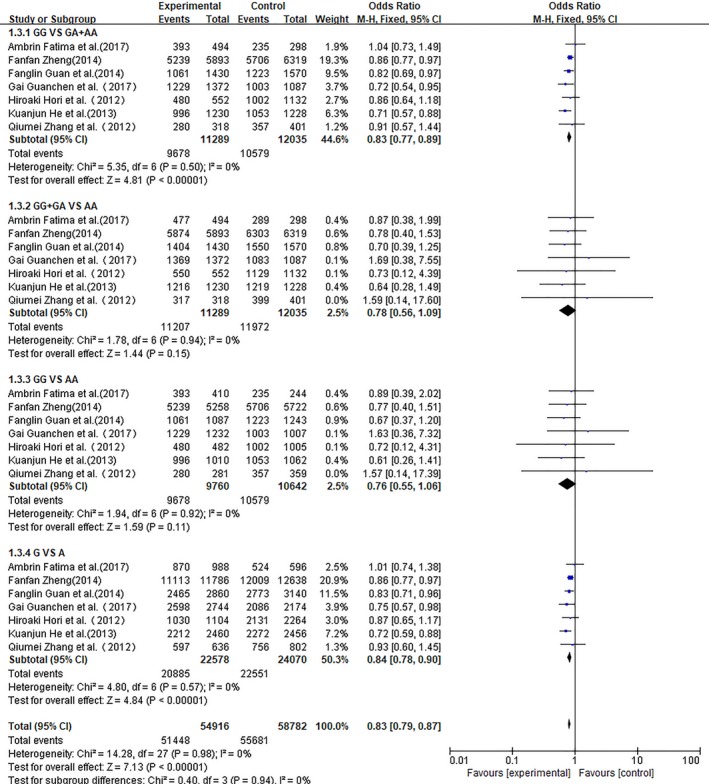
Meta‐analysis for the association of rs1006737 with schizophrenia in the Asian population

We conducted a meta‐analysis of Asian populations, European populations, and total populations. ORs with corresponding 95% CIs for the association between the rs1006737 polymorphism in the CACNA1C gene and the risk for schizophrenia in the different populations that were studied are detailed in Figures 2, 3, 4, respectively. In each meta‐analysis, the recessive model (GG vs. GA + AA), dominant model (GG + GA vs. AA), additive model (GG vs. AA), and allele model (G vs. A) were tested.

Nine studies including two European‐decent samples and seven Asian cohorts contributed 12,744 cases and 16,460 controls for the analysis of CACNA1C rs1006737 and schizophrenia. As no heterogeneity was detected under any genetic model (*p* > 0.05 and *I* ^2^ < 50%), the fixed effect model was applied to all the models. As shown in Figure 2, a statistically significant difference under four models (GG vs. GA + AA: OR: 0.84, 95% CI: 0.79–0.90 *p* < 0.00001; GG + GA vs. AA: OR: 0.79, 95% CI: 0.67–0.93 *p* = 0.004; GG vs. AA: OR: 0.76, 95% CI: 0.64–0.90, *p* = 0.001; and G vs. A: OR: 0.85, 95% CI: 0. 81–0.90, *p* < 0.00001) were observed by pooling the night included studies.

For European‐decent samples, only two studies including 1,455 cases and 4,425 controls were included. Using a fixed effect model, a significant difference was identified between patients and controls for the four models (GG vs. GA + AA: OR: 0.88, 95% CI: 0.77–0.99 *p* = 0.04; GG + GA vs. AA: OR: 0.79, 95% CI: 0.65–0.95 *p* = 0.01; GG vs. AA: OR: 0.76, 95% CI: 0.59–0.97, *p* = 0.03; and G vs. A: OR: 0.88, 95% CI: 0. 80–0.96, *p* = 0.006).

For Asian samples, seven studies were included in the meta‐analysis. We observed a significant difference between patients and controls for the recessive model and allele model (GG vs. GA + AA: OR: 0.83, 95% CI: 0.77–0.89, *p* < 0.00001; G vs. A: OR: 0.84, 95% CI: 0. 78–0.90, *p* < 0.00001) using a fixed effect model but the dominant model (GG + GA vs. AA: OR: 0.78, 95% CI: 0.56–1.09 *p* = 0.15) and additive model (GG vs. AA: OR: 0.76, 95% CI: 0.55–1.06, *p* = 0.11) showed no significant difference between patients and controls.

### 3.3 Sensitivity analysis

As no severe heterogeneity was observed and eligible studies were limited, sensitivity analysis was not performed.

### 3.4 PUBLICATION BIAS

The results of the publication bias test for the rs1006737 polymorphism are presented in Supplement Figures [Link], [Link], [Link] and Supplement Table S1. No publication bias was found in the group when assessed with the Egger test or Begg's funnel plot.

## 4 DISCUSSION

The potential role of neurodevelopmental disorder in schizophrenia has been suggested but is still ambiguous. A recent review has detailed a pivotal role of neurodevelopmental disorder in the pathogenesis of schizophrenia (Rund, 2018). CACNA1C is a known marker of neurodevelopment that plays an important role in schizophrenic pathophysiology (Bhat et al., 2012; Blake et al., 2010; Yin et al., 2014). In the current study, we investigated CACNA1C rs1006737 in patients with schizophrenia.

As we expected, the *p* value of the four models in the combined population (European and Asian population) were all <0.05. The results were replicated in the European population. As for the Asian population, only two models (GG vs. GA + AA and G vs. A) showed a significant difference with schizophrenia. Therefore, the overall meta‐analysis proves that there is a significant association between rs1006737 and schizophrenia, and allele A of rs1006737 is associated with the risk for schizophrenia at a comparable power within both populations. Our results are consistent with most previous studies (Jiang et al., 2015; Nie et al., 2015; Zheng et al., 2014).

Considering the difference in the minimum allele frequencies (MAF) in each study, ranging from 0.041 in Han Chinese populations to 0.333 in European populations, we conducted heterogeneity analysis. To our surprise, no heterogeneity was found in our meta‐analysis between European and East Asian ancestries. Similarly, heterogeneity analysis was performed on Asian and European populations separately, and the same results were obtained. In addition, our meta‐analysis showed no publication bias.

There are, however, limitations to the interpretation of our results. First, there are few studies included. Due to insufficient information provided in the original literature, several articles were not included in the meta‐analysis. Future research should include as much information as possible for more realistic results. Second, because the current research is only in Europe and Asia, the relationship between rs1006737 and schizophrenia in other ethnic groups cannot be determined. Therefore, there is an urgent need to conduct research on American, Oceanian, and African populations to understand the relationship between *CACNA1C* rs1006737 and schizophrenia in the world's populations.

Our findings contributed important evidence for the establishment of *CACNA1C* as a susceptibility gene for schizophrenia across world populations, but further investigations on its role in the pathogenesis of schizophrenia are warranted.

## CONFLICT OF INTEREST

The authors declare no conflict of interest.

## AUTHOR CONTRIBUTIONS

Dongjian Zhu, Jingwen Yin, and Chunmei Liang were responsible for the study design, statistical analysis, and manuscript preparation. Xudong Luo, Dong Lv, Zhun Dai, and Susu Xiong managed the literature searches and analyses. Jiawu Fu, You Li, and Juda Lin were involved in evolving the ideas. The study was supervised by Zhixiong Lin, Yajun Wang, and Guoda Ma.

## DATA AVAILABILITY STATEMENT

The data that support the findings of this study were derived from the following resources available in the public domain: Science Direct at https://www.sciencedirect.com/, Wiley Online Library at https://onlinelibrary.wiley.com/, PubMed at https://www.ncbi.nlm.nih.gov/pubmed, Cochrane Library at https://www.cochranelibrary.com/, and Wanfang data resource database at http://www.wanfangdata.com.cn/index.html. Additional datasets generated and analyzed during the current study are available from the corresponding author on reasonable request.
