# Supplementary material for: CACNA1C (rs1006737) may be a susceptibility gene for schizophrenia: An updated meta‐analysis
Source: Brain Behav. 2019 Apr 29;9(6):e01292. doi: 10.1002/brb3.1292 (PMC6576147; doi:10.1002/brb3.1292)
Supplement: Supplementary file 1 [file BRB3-9-e01292-s001.pdf]

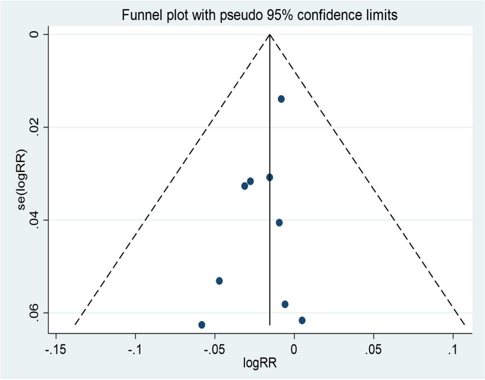


(A ) recessive model (GG versus GA + AA)

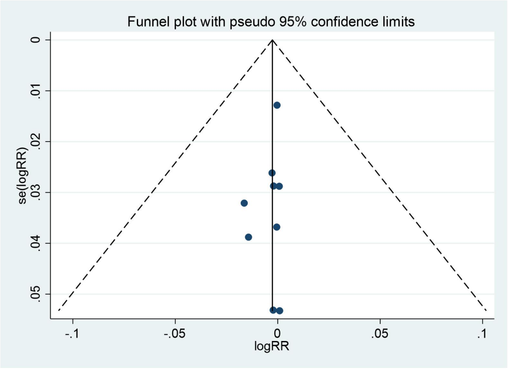


(B) dominant model(GG + GA versus

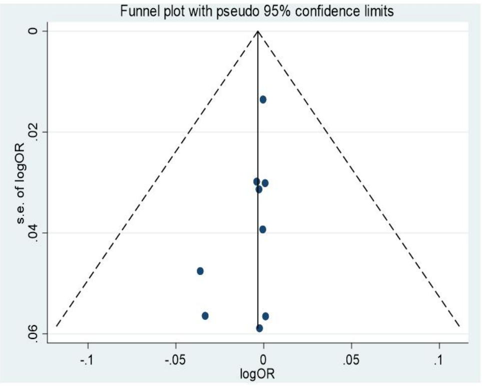


(C) additive model (GG versus AA)

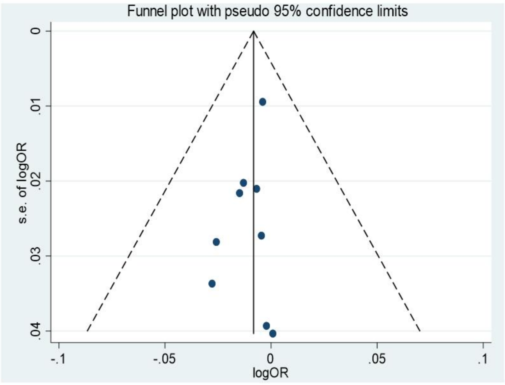


(D) allele model (G versus A)
