# Supplementary material for: CACNA1C (rs1006737) may be a susceptibility gene for schizophrenia: An updated meta‐analysis
Source: Brain Behav. 2019 Apr 29;9(6):e01292. doi: 10.1002/brb3.1292 (PMC6576147; doi:10.1002/brb3.1292)
Supplement: Supplementary file 4 [file BRB3-9-e01292-s004.docx]

Supplementary Material

**CACNA1C (rs1006737) may be a susceptibility gene for schizophrenia: an updated meta-analysis**

**Dongjian Zhu^1#^, Jingwen Yin^1#^, Chunmei Liang^2,3#^, Xudong Luo^1^, Dong Lv^1^, Zhun Dai^1^, Susu Xiong^1^, Jiawu Fu^2^, You Li^2,3^, Juda Lin^1^, Zhixiong Lin^1*^, Yajun Wang^4*,^ Guoda Ma^2,3*^**

*** Correspondence:**

Zhixiong Lin:zhixionglinzj@163.com

Yajun Wang: wangyajuny1977@aliyun.com

Guoda Ma: [sihan1107@126.com](mailto:sihan1107@126.com)

Supplementary Table 1 Egger's test for the publication bias

| Genetic Model | t For Egger's test | P For Egger's test |
| --- | --- | --- |
| combined European and Asian studies |  |  |
| recessive model  (GG versus GA + AA) | -1.61 | 0.152 |
| dominant model  (GG + GA versus AA) | -1.11 | 0.302 |
| additive model  (GG versus AA) | -1.65 | 0.142 |
| allele model  (G versus A) | -1.43 | 0.196 |
|  |  |  |
| European studies |  |  |
| recessive model  (GG versus GA + AA) | - | - |
| dominant model  (GG + GA versus AA) | - | - |
| additive model  (GG versus AA) | - | - |
| allele model  (G versus A) | - | - |
|  |  |  |
| Asian studies |  |  |
| recessive model  (GG versus GA + AA) | -0.51 | 0.634 |
| dominant model  (GG + GA versus AA) | -0.45 | 0.674 |
| additive model  (GG versus AA) | -0.56 | 0.597 |
| allele model  (G versus A) | -0.46 | 0.662 |

Supplementary Table 1 Egger's test for the publication bias
